# The Role of Methadone in Postoperative Analgesia in Esophagectomy Patients–A Retrospective Study

**DOI:** 10.3390/healthcare13172153

**Published:** 2025-08-29

**Authors:** Jesse Cheng, Emily Peng, Kaelan Wong, Ricki Pad, Natasha Mason, Mart Andrew Maravillas, Xueqin Ding

**Affiliations:** 1 School of Medicine, Case Western Reserve University, Cleveland, OH 44106, USA; jxc2050@case.edu (J.C.); exp231@case.edu (E.P.); kxw591@case.edu (K.W.); 2 Department of Anesthesiology, University Hospitals Cleveland Medical Center, Case Western Reserve University, Cleveland, OH 44106, USA; ricki.pad@uhhospitals.org (R.P.); natasha.mason@uhhospitals.org (N.M.); 3 Department of Biostatistics, University Hospitals Clinical Research Center, Cleveland, OH 44106, USA; martandrew.maravillas@uhhospitals.org; 4 Department of Anesthesiology, Metrohealth Medical Center, Case Western Reserve University, Cleveland, OH 44109, USA

**Keywords:** methadone, esophagectomy, postoperative pain, opioid consumption, multimodal analgesia

## Abstract

**Background:** Effective pain control is key to recovery after esophagectomy. Methadone may enhance analgesia and reduce opioid needs. Its role in thoracic surgery is not well defined. **Methods:** This single-center retrospective cohort study included 206 patients who underwent esophagectomy from 2017 to 2023. A total of 66 received intraoperative methadone, and 140 served as controls. The primary outcome was cumulative postoperative opioid use in morphine milligram equivalents (MMEs) at 12, 24, 36, 48, and 72 h. Secondary outcomes included pain scores, time to first opioid, and opioid-related side effects. **Results:** Demographics were similar between groups. Intraoperative opioid use was lower in the methadone group (49.9 ± 31.0 vs. 76.1 ± 39.6 MME, *p* < 0.001). Postoperatively, MME use was significantly lower in the methadone group at all time points: 12 h (38.4 ± 48.1 vs. 56.4 ± 53.5; *p* = 0.017), 24 h (76.7 ± 81.2 vs. 122.4 ± 109.7; *p* < 0.001), 36 h (103.8 ± 106.2 vs. 176.9 ± 156.9; *p* < 0.001), 48 h (139.4 ± 135.6 vs. 229.6 ± 210.7; *p* < 0.001), and 72 h (173.5 ± 173.5 vs. 304.0 ± 286.1; *p* < 0.001). Time to first opioid was longer (225.6 ± 296.5 vs. 41.6 ± 69.8 min, *p* < 0.001). Pain scores were similar in the first 72 h; at two weeks, they were lower with methadone (0.69 ± 1.42 vs. 1.43 ± 2.55, *p* = 0.009). Side effect rates were similar. **Conclusions:** Intraoperative methadone is associated with reduced postoperative opioid use without increasing side effects.

## 1. Introduction

Esophageal cancer is the seventh most prevalent and sixth deadliest cancer globally [1,2]. The primary curative treatment is esophagectomy with lymphadenectomy, often combined with neoadjuvant chemoradiotherapy. Esophagectomy is associated with significant postoperative pain, primarily due to muscular and intercostal nerve damage during the procedure [3,4]. Effective pain management is crucial not only for minimizing pulmonary complications and the risk of chronic postsurgical pain but also for enhancing patient comfort and improving recovery outcomes.

Over the past two decades, minimally invasive surgical techniques for esophagectomy have gained popularity. Approaches such as the McKeown procedure, which employs upper abdominal laparoscopy, right thoracoscopy, and cervical incision, as well as the Ivor Lewis procedure, which combines upper abdominal laparoscopy with right thoracoscopy, have become more common. These minimally invasive methods reduce tissue damage, leading to less postoperative pain and fewer pulmonary complications. However, despite these advancements, effective pain management remains essential to minimize complications and ensure patient comfort [5].

Traditionally, thoracic epidurals have been the standard method for managing pain after esophagectomy [6,7]. However, they are not suitable for patients on anticoagulation therapy and can fail due to misplacement, which may result from complex anatomy or postoperative catheter displacement [8,9]. Even when correctly placed, epidurals carry the risk of infrequent but severe complications such as neurological injury, epidural hematoma, and abscess [10]. Additionally, they can cause side effects like hypotension, motor block, and urinary retention, all of which can impede postoperative recovery [11]. Hypotension is particularly worrisome in esophagectomy patients as it can reduce blood flow to the gastric conduit, potentially extending the need for noradrenaline or increasing the risk of gastric conduit necrosis or anastomotic leakage [12]. Alternatives, like paravertebral [13] and erector spinae blocks [14], have been explored. However, some surgeons have reservations about using fascial plane blocks, citing concerns about interference with surgical exposure, variable local anesthetic spread leading to incomplete analgesia, and insufficient visceral analgesia for esophagectomy.

Methadone has gained attention for its unique dual action as both an NMDA receptor antagonist and a long-acting opioid agonist, offering potent pain relief. Its extended duration of action provides sustained pain control, reducing the need for frequent opioid dosing and avoiding the fluctuations in pain levels often associated with other opioids. Methadone’s efficacy has been well-documented across various surgical settings, including cardiac, abdominal, and spinal surgeries, with studies indicating that a single dose of methadone can improve pain scores and decrease opioid consumption by 40–50% without increasing the risk of side effects or complications [15,16].

Despite its promising benefits, the role of methadone in postoperative analgesia for thoracic surgeries, specifically esophagectomy, remains largely unexplored. We hypothesized that intraoperative methadone would be associated with reduced postoperative opioid consumption without an increase in opioid-related side effects. Specifically, we expected methadone to provide opioid-sparing effects and potentially longer time to first rescue analgesia, without increasing the incidence of adverse events. Our study aims to investigate the effect of methadone on pain management following esophagectomy, with the potential to enhance patient outcomes and establish new standards for postoperative care in this challenging procedure.

## 2. Methods

### 2.1. Study Design and Participants

Patients who underwent esophagectomy between 2017 and 2023 were identified using the electronic medical records (EMR) system database at University Hospitals Cleveland Medical Center. This retrospective analysis was approved by University Hospitals Cleveland Medical Center. This study’s retrospective design inherently limits causal inference, which we acknowledge in Section 2 and Section 4.

Inclusion criteria were as follows: (1) minimally invasive esophagectomy; (2) American Society of Anesthesiologists (ASA) physical status I–III; and (3) age 18 years or older. Exclusion criteria included: (1) patients with chronic pain; (2) those who received preoperative acetaminophen, gabapentin, and/or intraoperative ketamine, lidocaine, dexmedetomidine, and magnesium infusion during surgery; (3) patients undergoing open esophagectomy; and (4) patients who received regional anesthesia for postoperative pain control. These exclusions were made to isolate the effect of methadone but recognize that this narrows generalizability.

Data recorded included patient demographics (age, gender, BMI), ASA physical status, history of chemoradiation therapy, history of thoracic and abdominal surgery, duration of surgery, preoperative pain medication and sedation, intraoperative pain medication consumption, postoperative pain medication consumption at various time intervals (12, 24, 36, 48, and 72 h), minimum, average, and maximum pain scores within the first 72 h postoperatively, time to first pain medication administration, incidence of postoperative nausea and vomiting, opioid-related complications (such as respiratory depression, nausea/vomiting, and arrhythmias), length of ICU and hospital stay, and development of chronic surgical pain.

### 2.2. Interventions

All patients included in this study underwent either a McKeown or Ivor Lewis procedure. The decision to administer premedication to patients, when necessary, was entrusted to the judgment of the anesthesia care team. Once in the OR, general anesthesia was administered. Rapid sequence induction was performed using intravenous lidocaine (1–1.5 mg/kg), fentanyl (50–100 μg), propofol (1–2 mg/kg), and succinylcholine (1 mg/kg) or rocuronium (1–1.2 mg/kg). Intraoperatively, patients received either intravenous methadone (methadone group) and/or another opioid (control group) for pain management. At our institution, there are no specific protocols for pain control during esophagectomy, so the choice of methadone or other opioids was made by the anesthesiologist based on their experience and preferences. For patients in the Methadone group, a single intravenous bolus of 10, 15, or 20 mg methadone was given during or right after induction of anesthesia. Doses were fixed and determined at the discretion of the attending anesthesiologist, rather than weight-based. At our institution, fixed dosing was chosen for ease of administration and consistency, supported by prior literature demonstrating efficacy and safety in this range regardless of body weight.

All patients were intubated with a double-lumen endotracheal tube to facilitate single-lung ventilation, and anesthesia was maintained with sevoflurane and/or a propofol infusion. Intraoperative parameters such as hemodynamic status, pain medication usage, surgery duration, and any complications were carefully documented. At the end of the procedure, local anesthetic was infiltrated into the incision sites. As per routine practice, 4 mg of IV ondansetron was administered to each patient for antiemetic prophylaxis near the end of surgery. Postoperatively, patients were transferred to the surgical intensive care unit (SICU) for monitoring.

Upon transfer to the SICU, Patient-Controlled Analgesia (PCA) with hydromorphone was initiated (bolus dose of 0.2 mg, 10 min lockout, maximum of 1.2 mg per h). SICU nurses assessed pain severity at rest using an 11-point Numeric Rating Scale (NRS), where 0 indicated no pain and 10 represented the worst pain. Postoperative nausea and vomiting were documented and treated as needed. Once transferred to the general ward, patients received multimodal analgesia, including oral acetaminophen, oxycodone, tramadol, IV toradol, and either intravenous hydromorphone boluses or continued PCA. Pain scores were assessed at rest by SICU nursing staff according to standard protocol and were extracted directly from the electronic medical record. In our institution, postoperative monitoring includes continuous SpO_2_ and respiratory rate monitoring in the SICU for at least 24 h, with sedation scores documented every 2–4 h. After transfer to the general ward, SpO_2_ and respiratory rate are recorded every 4 h, with sedation scores assessed each nursing shift.

### 2.3. Outcome Measures

The primary outcome of this study was the cumulative opioid consumption at 12, 24, 36, 48, and 72 h postoperatively. This was calculated by summing all rescue opioid doses and total patient-controlled analgesia (PCA) usage, then converting them into Intravenous Morphine Equivalents (IME). Secondary outcomes included: (1) minimal, average, and maximal pain scores at 72 h postoperatively; (2) time to the first administration of pain medication (bolus of pain medication administered either by patient or nurse); (3) incidence of postoperative nausea and vomiting; (4) respiratory complications, including respiratory insufficiency (respiratory rate < 8/min), oxygen desaturation (<90%), excessive sedation, and reintubation; (5) QTc prolongation and other arrhythmias; (6) procedure-related complications such as anastomosis leak or conduit ischemia; (7) length of ICU and hospital stay; (8) pain scores and other complications at the first office visit, typically 2 weeks after surgery; and (9) Evaluation of chronic pain at 3 months postoperatively, including the use of scheduled opioids during this period. All conversions to morphine milligram equivalents were performed using standard equivalency ratios based on CDC 2022 guidelines.

### 2.4. Statistical Analysis

Continuous variables are presented as mean ± standard deviation, and categorical variables as counts and percentages. Between-group comparisons of continuous variables used the Student’s *t*-test when normally distributed and the Wilcoxon Mann–Whitney test when non-normally distributed, with normality assessed prior to test selection. Categorical variables were compared using Pearson’s chi-square or Fisher’s exact test when expected cell counts were low. Intragroup comparisons among methadone dose subgroups (10, 15, and 20 mg) employed the Kruskal–Wallis rank-sum test and Fisher’s exact test, as appropriate.

Effect sizes were reported as Cohen’s d for continuous variables and odds ratios (ORs) with 95% confidence intervals for categorical outcomes. For rare events (e.g., respiratory complications, atrial fibrillation), post hoc power calculations were performed based on observed proportions. Dose–response relationships were examined using a linear trend test across methadone subgroups. When only group-level data were available, approximate patient-level simulations assuming normal distributions with reported subgroup means and standard deviations were used to estimate trend significance, recognizing the exploratory nature of this approach. No adjustments for multiple comparisons were made due to the study’s exploratory intent. All statistical analyses were performed using R version 4.3.2, and a two-sided *p* < 0.05 was considered statistically significant.

In addition, using the Least Absolute Shrinkage and Selection Operator (LASSO) regression, we conducted a multivariable linear regression with 72 h cumulative postoperative opioid consumption (MME) as the dependent variable. Covariates included age, sex, body mass index (BMI), ASA physical status classification, procedure type, operative time, prior thoracic or abdominal surgery, neoadjuvant therapy, preoperative sedation, intraoperative opioid doses, body weight–adjusted methadone dose (mg/kg), and total postoperative doses of NSAIDs (ketorolac) and acetaminophen. The model was designed to minimize overfitting relative to the sample size and to estimate methadone’s independent association with postoperative opioid use.

## 3. Results

### 3.1. Participants

**Figure 1:**
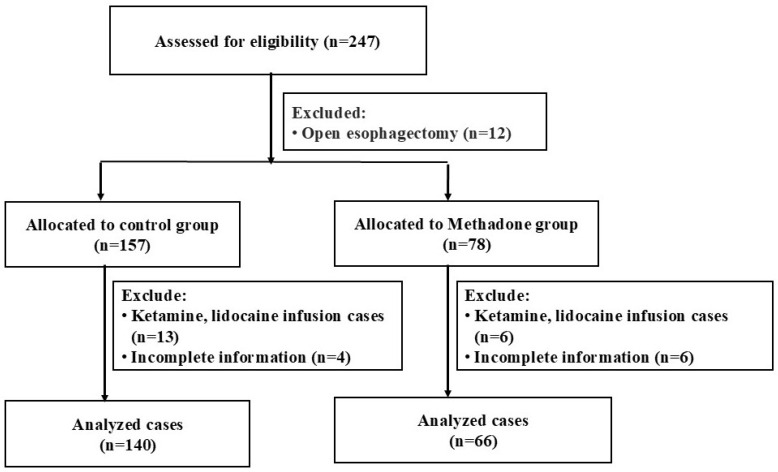
Flow Diagram for Retrospective Study.

A total of 247 patients who underwent esophagectomy were identified for the study. After excluding 12 patients who had open esophagectomy, 19 patients who received intraoperative ketamine, lidocaine, magnesium, or dexmedetomidine, and 10 patients due to missing documentation, the final analysis included 206 patients. These patients were divided into two groups: the control group (n = 144) and the methadone group (n = 66) (Figure 1). In the methadone group, 14 patients were administered 10 mg, 10 patients received 15 mg, and 42 patients were given 20 mg of methadone.

There were no significant differences between the Methadone and control groups regarding age, sex, ASA classification, BMI, preoperative pain medication use, sedation, neoadjuvant therapy, or a history of thoracic and/or abdominal surgery (*p* > 0.05, Table 1). Surgical duration was also similar between the two groups (*p* > 0.05). Intraoperatively, the Methadone group required significantly less pain medication than the control group (*p* < 0.05, Table 2).

### 3.2. Primary Outcome

The total morphine milligram equivalents (MME) for the methadone group at 12, 24, 36, 48, and 72 h postoperatively were 38.39 ± 48.13, 76.69 ± 81.18, 103.78 ± 106.17, 139.35 ± 135.57, and 173.52 ± 173.48, respectively. These values were significantly lower than those of the control group, which were 56.40 ± 53.46, 122.43 ± 109.70, 176.87 ± 156.92, 229.61 ± 210.73, and 303.95 ± 286.06, as shown in Table 3. Statistically significant differences were observed between the two groups at all five time points (*p* < 0.01). Effect size analysis showed small-to-moderate reductions in opioid consumption at 12 h (d = 0.35), moderate reductions at 24 h (d = 0.45), 36 h (d = 0.51), and 48 h (d = 0.47), and a moderate reduction in 72 h totals (d = 0.51).

In the methadone group, there were no significant differences in demographic or intraoperative data among the three subgroups, which were further analyzed based on the amount of opioid consumed intraoperatively. However, the time to first opioid administration was notably shorter in the 20 mg methadone subgroup compared to the others. Postoperative opioid consumption showed significant differences among the three subgroups at all five measured time points (*p* < 0.01). A clear dose-response effect was observed, with the 20 mg methadone subgroup requiring significantly less opioid than the 10 and 15 mg groups (*p* < 0.001), as shown in Table 4.

### 3.3. Secondary Outcomes

The time to first opioid administration was significantly longer in the methadone group compared to the control group (225.62 ± 296.53 min vs. 41.60 ± 69.83 min; *p* < 0.001), with a large effect size (d = –1.04). Pain scores within the first 72 h, SICU stay, and total hospital stay were similar between the two groups (*p* > 0.05). At the first postoperative clinic visit, however, the methadone group reported significantly lower pain scores (*p* = 0.009), exceeding the MCID threshold for NRS pain reduction. There were no differences in chronic opioid use at three months.

### 3.4. Dose–Response Analysis

Within the methadone group, demographic and intraoperative variables did not differ significantly among the three dose subgroups (10, 15, 20 mg). However, postoperative opioid requirements differed at all measured time points (*p* < 0.01), with the 20 mg subgroup consistently requiring the least. A dose–response trend was confirmed using simulated patient-level data derived from subgroup means and standard deviations, demonstrating a statistically significant negative slope (β ≈ –9.91 MME per mg; *p* ≈ 7.7 × 10^−7^).

### 3.5. Safety Outcomes

Rates of nausea and vomiting, respiratory depression (defined as SpO_2_ < 90%, respiratory rate < 8/min, or need for naloxone/reintubation), and QTc prolongation were similar between groups (all *p* > 0.05). Postoperative atrial fibrillation occurred in 17% of the control group and 29% of the methadone group (*p* = 0.044). None of the methadone group patients had preexisting atrial fibrillation, indicating new-onset cases. Subgroup analysis found no significant difference in atrial fibrillation rates among the methadone doses (*p* = 0.582). Other complications included anastomotic leak in six patients (four control, two methadone), conduit necrosis in two patients (one from each group), and one pulmonary embolus in the control group.

For categorical outcomes, odds ratios (OR, 95% CI, Fisher’s exact *p*) were: postoperative nausea/vomiting OR = 1.11 (0.41–3.03), *p* = 1.00; respiratory complications OR = 1.12 (0.48–2.59), *p* = 1.00; atrial fibrillation OR = 0.51 (0.26–1.02), *p* = 0.066. Post hoc power analysis indicated the study was underpowered to detect small differences in rare events: ≈0.055 for nausea/vomiting, ≈0.058 for respiratory complications, and ≈0.46 for atrial fibrillation.

### 3.6. Multivariable Analysis

LASSO regression was used to identify predictors of 72 h cumulative postoperative opioid consumption (TOPOC), with covariates including age, sex, BMI, ASA classification, procedure type, operative time, prior surgical history, neoadjuvant therapy, preoperative sedation, intraoperative opioid doses, body weight–adjusted methadone dose, and postoperative NSAID/acetaminophen use.

In the multivariable LASSO regression model (Table 5), intraoperative methadone remained a strong independent predictor of reduced 72 h postoperative opioid consumption (β = –298.6). Age was negatively associated with opioid requirements (β = –148.0), indicating that older patients required fewer opioids after esophagectomy. In contrast, higher BMI was associated with increased opioid use (β = +23.0 per unit increase). ASA physical status and operative duration were not retained as independent predictors after penalization, suggesting a limited impact once other variables were accounted for. Similarly, the use of multimodal agents, including NSAIDs and acetaminophen, did not significantly influence postoperative opioid consumption in this cohort. Overall, the model explained approximately 20.4% of the variance in opioid use, underscoring methadone and patient-specific factors (age and BMI) as the principal drivers of postoperative analgesic requirements. Based on its cross-validated R^2^, the final model explained approximately 20.4% of the variance in TOPOC. Intraoperative methadone use emerged as an independent predictor of reduced 72 h cumulative postoperative opioid consumption, associated with a mean reduction of approximately 299 MME after adjustment for all listed covariates (β = –298.59).

## 4. Discussion

Our study demonstrated that a single intraoperative dose of methadone significantly reduced opioid consumption in patients undergoing esophagectomy without causing notable side effects. However, we did not observe a significant difference in pain scores between the two groups during the first 72 h postoperatively, suggesting that methadone may be opioid-sparing without providing superior analgesia in the immediate postoperative period. This could reflect a ceiling effect of opioid efficacy or adequate analgesia from multimodal regimens in both groups. While opioid-related side effects such as nausea, vomiting, and sedation were comparable between groups, the substantial reduction in opioid use in the methadone group did not translate into statistically significant reductions in these complications. This may reflect multifactorial etiologies for postoperative nausea and vomiting and the impact of routine antiemetic prophylaxis.

Our multivariable analysis reinforces the robustness of the unadjusted findings by demonstrating that intraoperative methadone is independently associated with significantly lower postoperative opioid requirements, even after controlling for potential confounders such as age, BMI, ASA status, surgical duration, and use of multimodal agents. This highlights methadone’s opioid-sparing effect as not merely a reflection of intraoperative practice variability, but as a consistent pharmacologic benefit.

Managing postoperative pain following esophagectomy remains a significant challenge for clinicians, as regional anesthesia options like thoracic epidurals, paravertebral blocks, and erector spinae blocks are often limited by contraindications or surgical preferences. A systematic review shows no clear superiority of any single pain management approach [17]. At our institution, patient-controlled analgesia (PCA) is the standard for minimally invasive esophagectomy. However, PCA often leads to fluctuating drug levels, risking periods of insufficient pain relief or sedation. An ideal analgesic would provide steady, long-lasting pain relief without significant respiratory depression, enhancing recovery.

Methadone is an effective opioid alternative with a long half-life (24–36 h), providing stable blood levels from a single intraoperative dose and up to 35 h of analgesia at doses ≥20 mg [18,19]. It acts quickly in the CNS, reduces hyperalgesia and opioid tolerance through NMDA antagonism, and may aid postoperative mood stabilization by inhibiting serotonin and norepinephrine reuptake [20].

Two previous meta-analyses [15,21] found that the intraoperative administration of methadone significantly improved pain control by reducing opioid consumption compared to other opioids. These analyses encompassed studies from the 1980s to 2019, examining methadone’s effects across a range of surgical procedures, including cardiac, major spine, orthopedic, abdominal, urological, and gynecological surgeries. However, the role of methadone in thoracic surgeries, particularly esophagectomy, has been underexplored. In line with findings from other surgical contexts, our study demonstrated that a single dose of methadone not only decreased the use of intraoperative pain medication but also significantly reduced postoperative opioid consumption in esophagectomy patients.

While intraoperative methadone offers promising pain relief, safety concerns, particularly regarding severe respiratory depression, persist [22], which may explain the lack of studies in thoracic surgery. Potential adverse effects could require interventions like manual ventilation, naloxone, or reintubation [23]. However, multiple randomized trials report no significant differences in respiratory depression incidence between methadone and other opioids [24,25], and a large retrospective review found less than 0.5% of cases needed naloxone for serious respiratory depression [26]. Previous studies have reported that perioperative respiratory depression is more likely at doses exceeding 0.25–0.3 mg/kg, particularly in opioid-naïve patients, although the incidence with a single intraoperative dose remains below 1% [15]. In our study, instances of respiratory depression in the methadone group did not require naloxone or reintubation, with no significant differences between groups. Further large-scale trials are essential to fully clarify methadone’s safety profile.

The optimal methadone dosing strategy that offers prolonged pain relief without inducing respiratory depression remains uncertain and may vary depending on the surgical procedure. Previous clinical trials have utilized methadone doses ranging from 0.1 to 0.3 mg/kg, while some have adopted a fixed dose of 0.2 mg/kg or a 20 mg dose. However, it is often unclear whether these doses in the studies were based on ideal body weight or actual body weight. Additionally, more invasive surgeries might necessitate higher methadone doses [27], and differences in patients’ opioid tolerance levels could also affect the ideal dosage. At our institution, we typically administer a single methadone dose of less than 20 mg, as the minimal effective dose for esophagectomy is still unknown. Our study demonstrated a clear dose-response relationship: patients who received higher fixed doses of methadone (and thus, higher mg/kg doses, particularly among those with lower body weights) experienced greater reductions in postoperative opioid requirements. While fixed dosing offers simplicity in clinical practice, our results underscore the importance of considering patient body weight when selecting methadone doses to optimize pain control and minimize opioid requirements. Future studies could compare fixed versus weight-adjusted dosing strategies more directly to further refine methadone analgesia protocols.

Chronic methadone use or high doses have been linked to an increased risk of torsades de pointes, QT prolongation, and even sudden cardiac death [28]. However, the effect of a single intravenous dose of methadone on QT intervals and the potential for arrhythmias has not been extensively studied in randomized trials. Notably, clinical studies on perioperative methadone have not reported a higher incidence of adverse cardiac events. Similarly, clinically significant QT prolongation or arrhythmias are rare with single doses ≤0.3 mg/kg, with most cases of methadone-associated arrhythmia reported in the context of chronic high-dose therapy [15]. At our institution, we typically assess the patient’s QTc interval before deciding to administer methadone. In our study, we did not observe QT prolongation or other cardiac arrhythmias in the methadone group. While some patients did develop new-onset atrial fibrillation, we believe this was unrelated to methadone, as atrial fibrillation is a well-known complication following esophagectomy, probably due to stress and autonomic changes [29]. So far, there is no established mechanistic link to methadone.

Methadone, like ketamine, is an NMDA receptor antagonist that has been shown to reduce postoperative pain intensity. As a result, a single intraoperative dose of methadone may offer preventive analgesia and potentially lower the risk of developing chronic postsurgical pain. Komen et al. found that administering a small dose of methadone (0.15 mg/kg) during the anesthesia induction in ambulatory patients led to significantly lower pain levels at rest and reduced oral opioid requirements within the first 30 days postoperatively [30]. Despite the demonstrated short-term benefits, most research on perioperative methadone has not extended to long-term follow-up for chronic postsurgical pain. In our own study, although patients in the methadone group reported less pain two weeks post-surgery, the chart reviews did not reveal a statistically significant difference in pain scores between those receiving methadone and the control group three months postoperatively.

The role of methadone in Enhanced Recovery After Surgery (ERAS) protocols remains a topic of debate, with limited evidence available. Our study demonstrated that methadone significantly reduced opioid consumption in esophagectomy patients without increasing associated risks. Unchanged length of stay could reflect adequate pain control in both groups or a limited sample size to detect small differences. Collaborating with thoracic surgeons, we are evaluating methadone for potential inclusion in the ERAS protocol for esophagectomy at our institution; currently, its use remains investigational. Additionally, we plan to conduct a randomized controlled trial to further investigate methadone’s role in esophagectomy care.

In addition to confirming the independent association between intraoperative methadone and reduced postoperative opioid consumption, our multivariable analysis provided insight into other clinical predictors of opioid requirements. Increasing age was associated with markedly lower opioid use over 72 h, consistent with prior reports that older patients often have reduced analgesic requirements due to altered pain perception, pharmacokinetics, and greater opioid sensitivity [15]. Conversely, higher body mass index was associated with greater opioid consumption, which aligns with previous findings that patients with obesity may require higher perioperative opioid doses due to increased distribution volume, altered clearance, or challenges in pain control [31]. A history of abdominal surgery was associated with higher postoperative opioid requirements. This may reflect more complex surgical anatomy, adhesions, or altered pain sensitivity due to prior operative interventions. It suggests that surgical history can meaningfully influence postoperative analgesic needs. Patients who received preoperative sedation required substantially more opioids postoperatively. One possible explanation is that patients needing sedation may have had higher baseline anxiety, opioid tolerance, or increased perioperative analgesic needs, all of which can heighten postoperative opioid consumption. Receipt of neoadjuvant chemoradiation was associated with increased postoperative opioid consumption. This may be related to tissue fibrosis, heightened nociceptive response, or chemotherapy-induced neuropathic changes that amplify postoperative pain. It also underscores that oncologic treatment history can directly influence perioperative analgesic requirements. Greater intraoperative opioid administration was associated with higher postoperative opioid use. This is consistent with the idea that higher intraoperative opioid needs often reflect patients with higher baseline pain sensitivity or opioid tolerance, and may also reflect intraoperative practice patterns that set expectations for postoperative pain management. Notably, ASA physical status and operative duration were not retained as significant predictors, suggesting that baseline comorbidity burden and surgical length had less influence on postoperative opioid needs once methadone use and patient-specific factors were considered. Similarly, the use of adjunct multimodal agents such as acetaminophen and NSAIDs did not independently affect opioid requirements, likely reflecting their relatively consistent administration across the cohort. Together, these findings underscore the importance of tailoring perioperative analgesic strategies not only to surgical factors but also to individual patient characteristics such as age, BMI, and opioid pharmacodynamics.

This study has several limitations that should be taken into account when interpreting the results. Being a retrospective analysis, it is subject to inherent biases compared to randomized controlled trials. To minimize selection bias, we focused exclusively on patients undergoing minimally invasive esophagectomy. We excluded patients who received ketamine, dexmedetomidine, or magnesium infusions due to the lack of standardized protocols for these medications at our institution. Additionally, because the study relied on existing documentation in the electronic medical record (EMR), certain mild-to-moderate opioid-related side effects, such as pruritus and constipation, may not have been captured if they were not recorded. The patient’s mood status was also not documented, so we could not assess whether methadone had any effect on mood improvement. Additionally, pain scores were not consistently recorded at uniform time intervals, limiting our ability to compare pain levels across different time points. Lastly, as a single-center study, the findings may have limited generalizability; however, they provide valuable insights that could inform future research across diverse settings. Future research should aim to address these gaps by conducting prospective, randomized studies with standardized methadone dosing and pain assessment protocols to provide stronger evidence on its potential advantages over other opioids in the perioperative setting. Additionally, due to the retrospective EMR-based design, transient or mild adverse events such as short episodes of apnea, mild oversedation, or QT prolongation without clinical sequelae may not have been documented, which could lead to underestimation of adverse events.

## 5. Conclusions

Our study demonstrates that a single intraoperative dose of methadone was associated with reduced opioid consumption following esophagectomy. However, larger randomized controlled studies are required to further validate the safety and efficacy of methadone in thoracic surgical populations.

## Figures and Tables

**Table 1:** Preoperative data comparison between the control and methadone group.

	Group		*p*-Value ^2^
	Control, n = 140 ^1^	Methadone, n = 66 ^1^	
**Demographics**			
Age, years (Mean ± SD)	66.26 ± 10.20	66.53 ± 8.88	0.849
BMI, kg/m^2^ (Mean ± SD)	27.17 ± 5.47	28.24 ± 6.30	0.238
ASA, scale (Mean ± SD)	3.01 ± 0.22	3.02 ± 0.12	0.742
Sex (n, %)			0.740
Female	26.00 (18.57%)	11.00 (16.67%)	
Male	114.00 (81.43%)	55.00 (83.33%)	
**Preoperative Treatment/History**			
Sedation (n, %)	71.00 (50.71%)	38.00 (57.58%)	0.357
Neoadjuvant therapy (n, %)	105.00 (75.00%)	50.00 (75.76%)	0.906
Previous abdominal surgery (n, %)	91.00 (65.00%)	38.00 (57.58%)	0.304
Previous thoracic surgery (n, %)	19.00 (13.57%)	5.00 (7.58%)	0.211

^1^ n (%); Mean ± SD; ^2^ Welch Two Sample *t*-test; Pearson’s Chi-squared test; Fisher’s exact test.

**Table 2:** Intraoperative data comparison between control and methadone group.

	Group		*p*-Value ^2^
	Control, n = 140 ^1^	Treatment, n = 66 ^1^	
**Pain Medication**			
Fentanyl, MME	55.04 ± 34.62	39.32 ± 26.93	<0.001
Hydromorphone, MME	21.09 ± 15.73	10.55 ± 11.82	<0.001
Total opioid, MME	76.13 ± 39.56	49.86 ± 31.01	<0.001
**Surgery**			
Surgery time, min	405.96 ± 93.27	406.42 ± 116.64	0.978
QT prolongation and arrhythmia (n, %)	1.00 (0.71%)	0.00 (0.00%)	>0.999

^1^ n (%); Mean ± SD; ^2^ Welch Two Sample *t*-test; Pearson’s Chi-squared test; Fisher’s exact test.

**Table 3:** Postoperative data comparison between control and methadone group.

	Group		*p*-Value ^2^
	Control, n = 140 ^1^	Methadone, n = 66 ^1^	
**Time when first Opioid taken, min**	41.60 ± 69.83	225.62 ± 296.53	<0.001
Total opioid, MME *			
12th h	56.40 ± 53.46	38.39 ± 48.13	0.017
24th h	122.43 ± 109.70	76.69 ± 81.18	<0.001
36th h	176.87 ± 156.92	103.78 ± 106.17	<0.001
48th h	229.61 ± 210.73	139.35 ± 135.57	<0.001
72nd h	303.95 ± 286.06	173.52 ± 173.48	<0.001
**Postoperative Outcome**			
Stay in the ICU, days	2.73 ± 4.87	2.02 ± 3.37	0.225
Nausea/vomiting (n, %)	14 (10%)	6 (9.09%)	0.990
Respiratory complication (n, %)	21.00 (15.11%)	9.00 (14.06%)	0.845
Arrhythmia (n, %)	24.00 (17.27%)	19.00 (29.69%)	0.044
Other complications (n, %)	18.00 (12.95%)	6.00 (9.38%)	0.464
Stay in the hospital, days	9.21 ± 7.89	8.28 ± 4.32	0.278
On pain medication after 3 months (n, %)	6.00 (4.29%)	3.00 (4.55%)	>0.999
**Pain Level, Scale**			
Minimum	0.58 ± 1.05	0.33 ± 0.83	0.071
Maximum	7.64 ± 1.34	7.33 ± 1.60	0.176
Mean	4.04 ± 1.33	3.87 ± 1.48	0.428
After 2 weeks	1.43 ± 2.55	0.69 ± 1.42	0.009
After 3 months	0.38 ± 1.53	0.27 ± 0.97	0.635

* Cumulative sum; ^1^ n (%); Mean ± SD; ^2^ Welch Two Sample *t*-test; Pearson’s Chi-squared test; Fisher’s exact test.

**Table 4:** The effect of different doses of Methadone: preoperative, intraoperative, and postoperative data comparison.

		Methadone		*p*-Value ^2^
	10 mg, n = 14 ^1^	15 mg, n = 10 ^1^	20 mg, n = 42 ^1^	
Age, years	66.43 ± 8.02	67.70 ± 8.29	66.29 ± 9.44	0.874
BMI, kg/m^2^	27.73 ± 8.56	26.52 ± 4.84	28.79 ± 5.76	0.540
ASA, scale	3 ± 0	3 ± 0	3.02 ± 0.15	0.751
Sex				0.314
Female (n, %)	2 (14.29%)	0 (0%)	9 (21.43%)	
Male (n, %)	12 (85.71%)	10 (100%)	33 (78.57%)	
Sedation (n, %)	4 (28.57%)	10 (100%)	24 (57.14%)	0.001
Total intraop opioid, MME	55.93 ± 22.64	35.30 ± 16.52	51.31 ± 35.15	0.149
Surgery time, min	393.43 ± 124.26	347.50 ± 52.67	424.79 ± 121.72	0.133
Time when first opioid taken, min	265.71 ± 55.49	231.93 ± 29.27	143.00 ± 27.34	<0.001
Total Postoperative Opioid, MME *				
12th h	65.57 ± 49.07	34.04 ± 49.08	18.60 ± 24.69	0.004
24th h	132.00 ± 73.82	69.61 ± 83.42	29.00 ± 22.38	<0.001
36th h	168.43 ± 110.98	97.22 ± 106.39	40.80 ± 30.84	<0.001
48th h	237.61 ± 133.33	127.16 ± 133.87	52.95 ± 37.60	<0.001
72nd h	289.57 ± 145.39	157.29 ± 179.67	70.95 ± 63.66	<0.001
**Pain Level, Scale**				
Minimum	0.14 ± 0.36	0.10 ± 0.32	0.45 ± 0.99	0.363
Maximum	7.07 ± 2.09	7.80 ± 1.23	7.31 ± 1.51	0.656
Mean	3.52 ± 1.63	4.12 ± 1.14	3.92 ± 1.51	0.575
After 2 weeks	1.14 ± 1.83	0.30 ± 0.95	0.63 ± 1.35	0.348
After 3 months	0.71 ± 1.82	0.00 ± 0.00	0.19 ± 0.59	0.465

* Cumulative sum; ^1^ n (%); Mean ± SD; ^2^ Kruskal-Wallis rank sum test; Fisher’s exact test.

**Table 5:** Coefficients and parameters of the LASSO regression models.

Factor/Parameter	cv_Model_Opioid
Dependent variable, unit	TOPOC (MME)
Optimal penalty parameter, λ	26.63
Cross-validated R^2^	0.204
(Intercept)	327.73
**Baseline Characteristics**	
Age	−148.02
Sex (Male)	49.91
BMI	22.96
ASA	0
**Medical History**	
abdominal surgery	145.12
thoracic surgery	0
**Operative Factors**	
Preoperative sedation	318.86
Preoperative neoadjuvant	321.04
Intraoperative methadone	−298.59
Intraoperative total opioid	26.25
Intraoperative surgery time	0
**Postoperative NSAID/Acetaminophen**	0

## Data Availability

The data supporting this study’s findings are available on request from the corresponding author. The data are not publicly available due to privacy or ethical restrictions.
